# Looking at Burning Mouth Syndrome Brain: The Prevalence of Idiopathic Intracranial Hypertension Radiologic Signs in BMS Patients

**DOI:** 10.1111/joor.70017

**Published:** 2025-07-21

**Authors:** Michele Davide Mignogna, Noemi Coppola, Stefania Leuci, Mattia Sansone, Federica Canfora, Daniela Adamo, Roberto De Simone

**Affiliations:** ^1^ Department of Neuroscience, Reproductive and Odontostomatological Sciences, Oral Medicine Unit University of Naples Federico II Naples Italy; ^2^ Department of Neurology and Stroke Unit, Headache Centre “San Giuseppe Moscati” Hospital Aversa Italy; ^3^ Department of Neuroscience, Reproductive Sciences and Odontostomatology, Headache Centre University of Naples Federico II Naples Italy

**Keywords:** burning mouth syndrome, chronic idiopathic painful disorder, idiopathic intracranial hypertension

## Abstract

**Background:**

Idiopathic intracranial hypertension (IIH), a disorder characterised by an elevation of intracranial pressure, has implications in chronic pain syndromes, especially in the cranial territory, and has been a matter of discussion. This study explores an association between burning mouth syndrome (BMS) and cerebrospinal fluid dynamic disturbances, as in IIH, by analysing the prevalence of MRI signs of impaired intracranial pressure control in BMS patients.

**Methods:**

This was a case–control with a cross‐section design study carried out at the Oral Medicine Unit, Federico II University of Naples, between September 2022 and March 2023. The observer was blinded on MRI measurements. Patients were recruited sequentially in the BMS referral centre of the Oral Medicine Unit. Thirty‐seven BMS patients and 37 healthy controls were consecutively enrolled during their first visit with an oral medicine specialist and a general dentist respectively. A non‐contrast brain MRI including an MRI venography of the intracranial venous vessels and a fundus oculi in order to evaluate the presence of papilloedema (an ophthalmological sign of raised intracranial pressure) was performed on all participants. The radiologic diagnostic criteria of IIH to include the patient in the study were at least 3 out of 4 indirect signs on the neuroimaging (empty sella, enlargement of the optic nerve sheets, bulb flattening and dural sinus stenosis).

**Results:**

Thirty‐seven patients with BMS and 37 HCs were included in the study with a female predominance in the BMS group. The prevalence of enlargement of the optic nerve sheath diameter (6.0 vs. 5.3; *p* < 0.001), the prevalence of empty sella (54.1% vs. 13.5%; *p* < 0.001), and the prevalence of dural sinus stenosis/hypoplasia (97.3% vs. 27%; *p* < 0.001) were statistically significantly higher in the BMS group than in HCs.

**Conclusions:**

The higher prevalence of IIH signs on neuroimaging (empty sella, enlargement of the optic nerve sheets and dural sinus stenosis) in BMS patients compared to HCs highlights that BMS should be considered a chronic neurological disorder. The investigation of CSF dynamics in BMS patients could reveal new perspectives on the diagnosis and pathogenesis of the disease.

## Graphical abstract

Prevalence of radiologic signs of idiopathic intracranial hypertension in burning mouth syndrome patients.

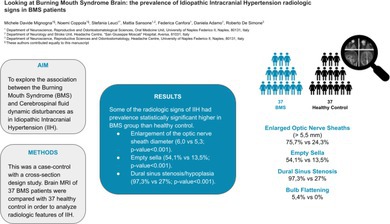

## 1 Introduction

Idiopathic intracranial hypertension (IIH) is a disorder characterised by an elevation of intracranial pressure (ICP). This condition is more common in overweight women in childbearing age; however, the gender preference and its pathophysiological relation to obesity are still unknown [1]. IIH can present with or without papilloedema. The IIH with papilloedema has a prevalence that has been estimated to be about 0.5–2 per 100.000 in the general population [2], reaching a worldwide incidence of around 12–20 per 100.000 people per year in the group of population between 25 and 36 years [3]. The IIH without papilloedema (also known as IIHWOP) instead is considered an uncommon kind with unknown prevalence so far, although there are several controversial data on this matter. Indeed, IIH may occur without its typical signs and symptoms (i.e., headache, visual disturbances and papilloedema) even it may run asymptomatically or present unexpectedly as chronic headache [4], fibromyalgia [5], stabbing headache or associated with physical effort [6, 7]. Moreover, it is widely demonstrated that there is an elevated intracranial pressure in about 11% of patients with bilateral dural sinus stenosis (a well‐known radiologic sign of IIH) and no other signs or symptoms of IIH, and an extraordinarily high prevalence of IIHWOP in an unselected series of patients with chronic migraine (10%–14%) [8] and refractory chronic migraine (22.5%–86.4%) [9, 10]. Based on these findings, it has been shown that the prevalence of IIH without papilloedema is approximately 1000‐fold greater than that of the IIH with papilloedema [4, 11]. This assumption enforces the idea that there is a clinical and epidemiological spectrum of IIH [12].

The pathogenesis of IIH is still unclear and several mechanisms have been proposed over decades, although a raised dural sinus pressure seems to be crucial for its physiopathology [13] explaining the very recurring presence of sino‐venous stenosis found in these patients [14] and the therapeutic effect of the dural sinus stenting [15]. Besides, an impairment of the sino‐venous system can lead to a subsequent overflow of the glymphatic system (GS), spoiling other cerebrospinal fluid (CSF) excretion routes, such as the meningeal lymphatic network and the peripheral nerves sheaths [16, 17].

Patients with IIH usually complain of headaches, vestibular and visual disturbances. These symptoms are typically worse in postures and actions that cause an increase in ICP, such as the supine position and Valsalva manoeuvre [18]. Increasingly over time, IIH has been connected to other chronic syndromes, such as mild cognitive disturbance and chronic pain syndromes (i.e., fibromyalgia and chronic fatigue syndrome) [19]. Besides, in the literature there are some connections among IIH and orofacial pain syndromes, such as a case report that demonstrated an association between IIH and persistent idiopathic facial pain [20]. Researchers have hypothesised that the irritation and/or compression of the fibres of the nerve roots caused by the increase in CSF pressure seen in these patients could explain these associations [5]. Thus, given the possibility of IIH to impair the peripheral nerves, including the cranial nerves, we hypothesised that IIH might be part of the physiopathological mechanism of the burning mouth syndrome (BMS) [21].

BMS is a disease characterised by a generalised or localised intraoral burning, dysesthetic sensation or pain of the oral mucosa (a trigeminal nerve territory) recurring daily for more than two hours per day for more than three months, without any clinical signs [22]. The worldwide prevalence of BMS is estimated to be 1.73% in the general population with a higher prevalence in middle‐aged and older women than the male population, with a female to male ratio of 3:1 [23]. BMS patients often reported co‐occurrence of intraoral dysesthesia and extraoral chronic painful comorbidities, such as ocular, otorhinolaryngological, neurological, cardiological, gastrointestinal and dermatological symptoms [24]. As stated above, IIH may overlap with fibromyalgia and chronic fatigue syndrome [20]. Besides, the role of the cranial nerve sheaths in the discharging of CSF from the subarachnoid space and GS [16] supports our hypothesis, where the raised CSF pressure might impair them, leading to a small fibre neuropathy as a result, as seen in many BMS patients, suggesting a likely overlap between them [25].

Finally, the association between IIH and BMS has not been explored. On these bases, the aim of this controlled clinical study is to evaluate the prevalence of IIH in BMS patients in an outpatient clinic of a university hospital through the investigation of the neuroimaging signs.

## 2 Results

### 2.1 Demographics

A total of 74 subjects were included in this study, 37 patients with BMS and 37 HC, and no missing data were recorded (Table 1). Regarding gender, there is a statistically significant difference between the two groups. In fact, expectedly, in the BMS group, there was a female predominance (32; 86.5%), while in the control group males prevailed (26; 70.3%) (*χ* ^2^(1) = 24 482, *p* < 0.001). The mean age of the BMS patients was 55.9 years. Mean age of the HC was 35.7 years. None of the included patients had papilloedema at fundoscopic examination.

**TABLE 1:** Sociodemographic characteristics and neuroimaging signs of the study group.

	BMS patients	Healthy controls	*p*
Gender, M/F	5/32	26/11	
Age, years	55.9	35.7	
Empty sella [*n* (%)]	20 (54.1%)	5 (13.5%)	< 0.001
Optic nerve sheath diameter^a^ [mean (standard deviation)]	6.0 (0.68)	5.3 (0.29)	< 0.001
Transverse sinus stenosis [*n* (%)]	36 (97.3%)	10 (27%)	< 0.001
Right [*n* (%)]	5 (13.9%)	5 (50%)	
Left [*n* (%)]	17 (47.2%)	5 (50%)	
Bilateral [*n* (%)]	14 (38.9%)	0	
Flattened posterior sclera [*n* (%)]	2 (5.4%)	0	> 0.05

^a^ The Levene's test and the *t*‐test have been used to assess the equality of variances for the variable ONSD.

### 2.2 Neurological Imaging

Brain MRIs of all subjects included in the study did not show alterations that could cause a secondary intracranial hypertension.

#### 2.2.1 Optic Nerve Sheath Diameter

BMS patients presented statistically significant higher average median total scores of the diameters of the optic nerve in comparison to the HC (6.0 vs. 5.3). In particular, a comparison between the BMS group and the control group showed that there was a significant difference in ONSD scores between the two groups (*p* < 0.001) (Table 1).

#### 2.2.2 Empty Sella

A statistically significant difference was observed in the prevalence of ES in the BMS patients and in the control group, with the first group reporting a higher prevalence of ES (*χ* ^2^(1) = 13.592, *p* < 0.001) (Table 1).

#### 2.2.3 Bulb Flattening

The majority of BMS patients (35; 94.6%) did not show bulb flattening; none of the HCs showed bulb flattening. No statistically significant difference was found between the two groups regarding the frequency of bulb flattening (*χ* ^2^(1) = 2.056, *p* > 0.05) (Table 1).

#### 2.2.4 Dural Sinus Stenosis/Hypoplasia

Statistically significant difference in the frequency distribution was detected when analysing the dural sinus stenosis with a higher prevalence in BMS group (36; 97.3%) compared with the control group (10; 27%) (*χ* ^2^(1) = 38.839, *p* < 0.001). To regard the site of the stenoses, in BMS patients left and bilateral stenosis prevailed (47.2% and 38.9%, respectively). In HCs, none showed bilateral stenosis. There was a significant correlation between the group and the site of the stenosis (*χ* ^2^(2) = 8.596, *p* < 0.005) (Table 1).

### 2.3 Frequency of Oral Symptoms and Site of BMS Patients

The frequency and the location of the oral symptoms for BMS patients are shown in Table 2. Oral burning was found in 37 patients (100%); of these, in six patients (16.22%) oral burning was found as a single symptom. Thirty‐one patients (83.78%) reported at least two additional oral symptoms. The most frequently involved sites in patients who complain of burning were tongue (30 pts.; 81.08%), lips (24 pts.; 64.86%) and palate (20 pts.; 54.05%).

**TABLE 2:** Frequency of oral symptoms and site of BMS patients.

	Frequency (%)
Oral symptoms	
Burning	37 (100)
Xerostomia	23 (62.16)
Dysgeusia	17 (45.95)
Shialorrhea	4 (10.81)
Itching	2 (5.40)
Subjective halitosis	4 (10.81)
Itraoral foreign body sensation	6 (16.22)
Oral dyskinesia	5 (13.51)
Occlusal dysesthesia	6 (16.22)
Globus sensation	14 (37.84)
Site	
Tongue	30 (81.08)
Lip	24 (64.86)
Palate	20 (54.05)
Gum	5 (13.51)
Cheeks	17 (45.95)
Floor of the mouth	6 (16.22)

## 3 Discussion

In this study, we investigated the prevalence of IIH in BMS patients. To the best of our knowledge, this is the first study that explores whether IIH could be associated with BMS. IIH is a pleiotropic condition. In the literature, it has been related to several manifestations, such as chronic headache, fibromyalgia and vestibular symptoms [20]. We reported a significant prevalence of IIH radiological signs in BMS patients compared to HCs. In fact, the analysis of the neuroimaging signs showed a statistically significant difference in the prevalence of pathological signs between the two groups. ONSD values of > 5.5 mm, indicating IIH, were found in most of our participants (75.7%) compared to HCs (24.3%).

Moreover, 54.1% of BMS patients showed ES versus 13.5% in the control group. Similarly, in 97.3% of BMS MRI dural sinus stenosis was detected compared with 27% in the control group. Finally, the frequency of bulb flattening in the two groups does not reach enough statistical significance (*p* > 0.05). In this regard, it is necessary to specify that the flattening of the posterior globe among the signs of IIH is the one with less reliability due to the influence of the orientation of the images [18]. As stated above, papilloedema is a required diagnostic sign of IIH. However, some cases, possibly much more frequent than currently believed, can present without it [4, 11]. In our cohort, none of the enrolled patients showed papilloedema at fundoscopic examination.

Based on the controversial revised IIH criteria, an IIH without papilloedema diagnosis, even ‘suspected’ requires the demonstration of an OP > 250 mm H_2_O [17]. Nevertheless, there is evidence that a combination of any 3 of 4 MRI features is highly specific for intracranial hypertension and suggests IIHWOP in patients lacking papilloedema. Based on neuroradiologic findings [17, 18], 51.3% of our BMS patients share at least 3 of 4 typical radiologic IIH signs and are therefore most probably IIHWOP carriers [18]. IIH is still a disease without a defined cause, although there are several theories. An increased production of cerebrospinal fluid (CSF) has been proposed, but evidence is lacking [26]. Instead, an impaired CSF circulation due to a derangement of the sino‐venous system may be a key factor [16]. Indeed, a raised venous pressure can slow down CSF reabsorption, causing elevation of the intracranial pressure [27, 28]. Besides, it has been suggested that a poor venous outflow due to dural sinus stenosis might be the firestarter (and not the effect) for exhausting the intracranial compliance, promoting large fluctuations of intracranial pressure to pathologic value [4].

The pathogenesis of BMS is still controversial. However, several studies have shown that the main mechanisms responsible for the onset of the pathology include peripheral and central neuropathy, and psychosocial components [21, 22, 23, 24, 25, 26, 27, 28, 29]. To regard central neuropathy, it is widely demonstrated that alterations in the medial system of the pain‐related network, structural and functional deficits in the medial prefrontal cortex and hippocampus, and changes in grey matter structure and white matter structure are present in BMS patients [21]. Moreover, growing evidence has shown the crucial role of peripheral neuropathology in BMS with a significant loss of epithelial and subepithelial nerve fibres together with an increased expression of the receptor potential subfamily member V1 (TRPV1) ion channel, and upregulation of the P2X3 receptors and of nerve growth factor (NGF) [30]. To date, data on a relationship between IIH and BMS are not available in literature. However, it has been suggested that an increased pressure in the subarachnoid spaces, with subsequent compression of the nerve roots, might cause radicular pain, as might happen in fibromyalgia [31]. The GS largely contributes to the overall CSF excretion rate through the meningeal lymphatic network and through the cranial and spinal nerve sheath [16]. It has been proposed that IIH is associated with a GS overflow involving the optical, the olfactory and other cranial nerves [16]. These considerations and findings might connect the IIH with the peripheral neuropathy seen in BMS patients [16]. BMS has been increasingly linked to peripheral neuropathy, particularly small fibre neuropathy, which affects unmyelinated C fibres and thinly myelinated Aδ fibres responsible for nociception and thermal perception. Evidence suggests that in many BMS patients, there is a dysfunction or degeneration of these peripheral sensory fibres in the oral mucosa, leading to chronic pain and dysesthesia in the absence of visible clinical signs [16]. Our data seem to cast a new hypothesis for the pathogenesis of BMS. In these patients, there might be an intracranial volume‐pressure impairment (as suggested by radiologic features) leading to a GS overflow manifesting as trigeminal dysfunction—related to BMS symptoms.

However, some limitations in our study should be noted. We did not perform lumbar puncture to assess the opening pressure of our patient series, and IIHWOP diagnosis has been proposed only based on typical IIH neuroradiologic findings. The series was quite small, albeit the findings clearly differentiated patients from controls. We analysed only the neuroimaging findings in the absence of tools to investigate the psychological profile in both groups. This study is a preliminary investigation; further studies with an increased number of patients enrolled, including OP assessment and response to treatments aimed at ICP control, are needed to confirm the hypothesis of the possible association between IIH and BMS.

In conclusion, the higher prevalence of IIH signs in BMS patients compared to controls confirms that BMS should be considered a chronic neurological disorder. The associations between CSF dynamics and BMS suggest new perspectives on the pathogenesis and diagnosis of BMS. Once the role of IIH in the pathogenesis of BMS has been clarified, future research could focus on potential new therapeutic approaches in the management of BMS.

## 4 Materials and Methods

### 4.1 Study Design

This was a case–control with a cross‐section design study carried out at the Oral Medicine Unit, Federico II University of Naples, between September 2022 and March 2023. The study protocol was approved by the local Ethics Committee (No. 125/19). It was conducted according to the guidelines of the World Medical Association Declaration of Helsinki (World Medical Association, 2013) and follows the Strengthening of the Reporting of Observational Studies in Epidemiology (STROBE) guidelines for the reporting of observational studies.

### 4.2 Participants

All potentially eligible participants were invited to participate in the study. The general procedure and requirements were explained and written informed consent was obtained from all participants. BMS patients were consecutively enrolled during their first visit with an oral medicine specialist. Eligible participants were identified based on the following inclusion and exclusion criteria.

The inclusion criteria for the BMS group were:

1. Male/female, > 18 years old
2. Diagnosis of BMS according to the International Classification of OroFacial Pain criteria for the study group [32]
3. No abnormalities in laboratory findings
4. No history of headache, vestibular or psychiatric disorder
5. No previous neurological consultation

The inclusion criteria for healthy controls were:

1. Male/female, > 18 years old
2. Patients without a history of BMS
3. No abnormalities in laboratory findings
4. No history of psychiatric disorder
5. No previous neurological consultation

The exclusion criteria for both groups were:

1. < 18 years old
2. Systemic disorders or laboratory abnormalities known to be potentially associated with BMS
3. Previous neurological consultation or ongoing treatment for symptoms associated with IIH
4. Use of psychotropic drugs in the past 3 months
5. Inability to understand or complete the questionnaires
6. Patients with diagnosis of other chronic pain

### 4.3 Procedure

The sociodemographic details, medical data and BMS characteristics of the patients were recorded during a standardised interview. Each participant to the study underwent a conventional oral examination to rule out other oral diseases. After the baseline examination, MRI scans of the brain were performed on all participants. Moreover, ophthalmological evaluation to rule out papilloedema was performed.

### 4.4 MRI of Brain

**FIGURE 1:**
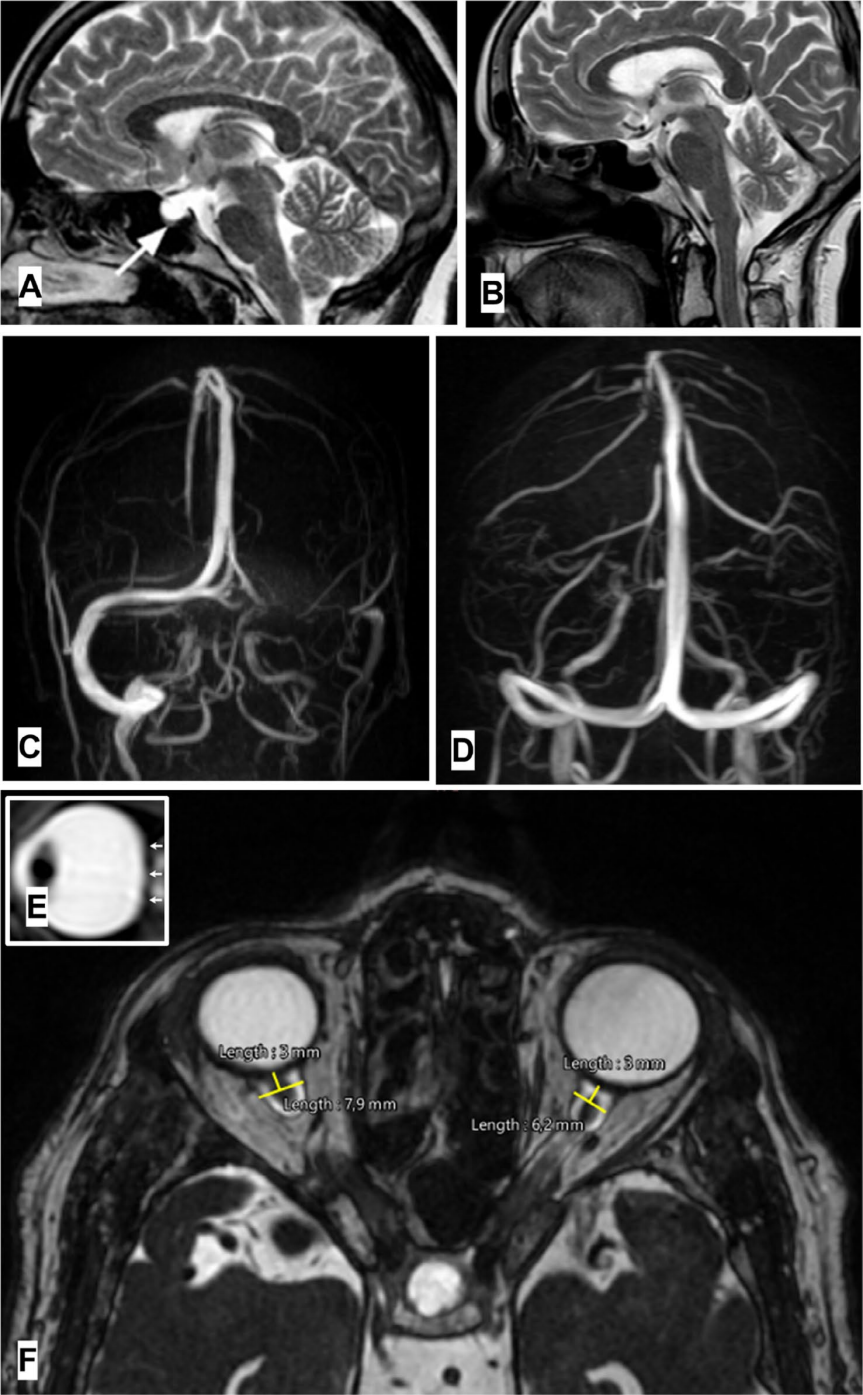
Indirect MRI sign of IIH: Empty sella (A) versus normal sella (B); dural sinus stenosis (C) versus normal dural sinuses (D); bulb flattening (E); enlargement of optic nerve sheaths (F).

MRIs were conducted in different labs and included T1‐ and T2‐weighted scans with fluid‐attenuated inversion recovery (FLAIR) with a section thickness of 3 mm. Scans were assessed by the same neurologist (M.S.). The diagnostic criteria of IIH are: opening pressure at lumbar puncture more than 250 mm H_2_O; evidence of papilloedema at fundoscopic examination or the presence of abducens nerve palsy; normal neurologic examination (except for the VI cranial nerve palsy); normal CSF composition and a normal neuroimaging (except for indirect signs of IIH) [17]. Without papilloedema/abducens nerve palsy, the diagnosis can be *suggested* providing at least 3 out of 4 indirect signs on the neuroimaging (empty sella [ES], enlargement of the optic nerve sheets, bulb flattening and dural sinus stenosis) (Figure 1) [18]. According to the diagnostic criteria of IIH reported above, the following MRI findings were evaluated: optic nerve sheath diameter (ONSD), ES, flattening of the posterior aspect of the optic globe and transverse sinus stenosis [18].

- The ONSD was measured on the axial T2 section 3 mm from the optic bulb as the maximum width of each optic nerve sheath. If the mean of the diameters was > 5.5 mm, the ONSD was enlarged.
- ES is referring to the arachnoid space invading the sella turcica and thus compressing the pituitary gland. The height of the pituitary gland was obtained on T2 mid‐sagittal scan, thus a measure < 4.8 mm was defined as ES.
- Bulb flattening of the posterior aspect of the optic globe was searched on T2 axial scan, where the optic nerve arises from the eye.
- The transverse sinus (TS) stenosis/hypoplasia was measured using the modified venous conduit score on a scale of 0–2 (0 = < 25% stenosis, 1 = 25%–50% stenosis and 2 = > 50% stenosis; 0 = < 25% smaller than the contralateral side, 1 = 25%–50% smaller than the contralateral side and 2 = > 50% diffuse narrowing compared with the contralateral side). Notably, angiographic studies have shown that a stenosis of 30% of the TS is sufficient to generate a clinically significant pressure gradient [33].

### 4.5 Statistical Analysis

The statistical analysis was performed using the SPSS software v. 26. Descriptive statistics, including means, SDs, medians and interquartile range (IQR), were used to analyse the sociodemographic and clinical characteristics of the two groups. The Pearson Chi‐Squared test was used to test the significance of differences between the percentages in the two groups. The Levene's test and the *t*‐test have been used to assess the equality of variances for the variable ONSD and then the difference between the two groups. Due to the sample size exceeding 30 in each group, the assumption of normality was considered met based on the Central Limit Theorem (CLT). Therefore, a parametric independent samples *t*‐test was conducted to compare the group means.

Differences associated with the values of *p* < 0.05 or 0.01 were considered respectively. For all statistical analyses, a significance level of 0.05 was adopted.

## Author Contributions

Conceptualization, M.D.M. and R.D.S.; methodology, M.S. and N.C.; software, F.C. and D.A.; validation, N.C. and S.L.; formal analysis, M.S. and C.N.; investigation, M.D.M., N.C., R.D.S.; resources, S.L. and D.A.; data curation, N.C. and M.S.; writing – original draft preparation, M.D.M., M.S. and N.C.; writing – review and editing, S.L. and D.A. All authors gave final approval and agreed to be accountable for all aspects of the work in ensuring that questions relating to the accuracy or integrity of any part of the work are appropriately investigated and resolved.

## Ethics Statement

The study protocol was approved by the local Ethics Committee (No. 125/19).

## Conflicts of Interest

The authors declare no conflicts of interest.

## Peer Review

The peer review history for this article is available at https://www.webofscience.com/api/gateway/wos/peer‐review/10.1111/joor.70017.

## Data Availability

The data that support the findings of this study are available from the corresponding author on reasonable request.
